# Predictors of change differ for moderate and vigorous intensity physical activity and for weekdays and weekends: a longitudinal analysis

**DOI:** 10.1186/1479-5868-10-69

**Published:** 2013-05-28

**Authors:** Kirsten Corder, Christopher Craggs, Andrew P Jones, Ulf Ekelund, Simon J Griffin, Esther MF van Sluijs

**Affiliations:** 1 UKCRC Centre for Diet and Activity Research (CEDAR), Institute of Public Health, University of Cambridge, Box 296, Forvie Site, Robinson Way , Cambridge CB2 0SR, UK; 2 MRC Epidemiology Unit, Institute of Metabolic Science, Box 285, Addenbrooke’s Hospital, Hills Road, Cambridge CB2 0QQ, UK; 3 Norwich Medical School, University of East Anglia, Norwich NR4 7JT, UK; 4 Department of Sports Medicine, Norwegian School of Sports Sciences, PO Box 4014, Ullevål Stadion, Oslo 0806, Norway

**Keywords:** Longitudinal, Behaviour change, Childhood, Determinants, Physical activity, Predictors

## Abstract

**Background:**

Predictors of physical activity (PA) change are rarely investigated separately for different PA intensities and for weekdays/weekends. We investigated whether individual-level predictors of one-year change in objectively-measured physical activity differ for moderate PA (MPA) and vigorous PA (VPA) and for weekends and weekdays.

**Methods:**

Accelerometer-assessed PA (mins) was obtained at baseline and +1 year (n = 875, 41.5% male, Mean ± SD baseline age: 9.8 ± 0.4 years-old). Potential predictors (n = 38) were assessed at baseline from psychological (e.g. self-efficacy), socio-cultural (e.g. parent support) and environmental domains (e.g. land use). Associations between predictors and change in MPA (2000–3999 counts/minute (cpm)) and VPA (≥4000 cpm) separately for weekdays and weekends were studied using multi-level linear regression. Analyses were adjusted for school clustering, sex and baseline PA.

**Results:**

Weekend PA declined (MPA decline 4.6 ± 21.8 mins/day; VPA decline: 2.1 ± 20.1 mins/day; both p < 0.001) whereas weekday PA did not significantly change. Higher baseline PA and being a girl were associated with greater PA declines in all four outcomes; remaining predictors differed for MPA and VPA and/or weekdays and weekends. Family logistic support was associated with less of a decline in weekend MPA (CI 95%) 0.15 (0.05, 0.25) and VPA 0.19 (0.09, 0.29), and peer support with less of a decline in weekday MPA 0.18 (0.02, 0.34) and VPA 0.22 (0.06, 0.38).

**Conclusions:**

Results highlight the relevance of investigating predictors of PA change separately for different PA intensities and for weekdays/weekends. In addition to continued focus on school PA promotion, more effort to target interventions during weekends, such as in the family and community appears important. Encouraging peer support to increase weekday PA and targeting parent support for weekend PA may be health promotion priorities.

## Background

Physical activity in children has been associated with reduced risk of the metabolic syndrome [1,2] and beneficial effects on mental health [3]. Physical activity appears to decline with age throughout childhood and adolescence [4,5] and inactive children may become inactive adults [6], resulting in a higher risk of health complications later in life [7]. Although increasing physical activity among young people is a public health priority, few interventions have been shown to effectively increase physical activity among children [8-10].

Knowledge on potentially modifiable factors influencing physical activity is important to inform physical activity promotion strategies [11]. However, a review of predictors of change in physical activity identified few consistent factors with which to inform intervention development [12]. These inconsistent associations could be at least partly due to differential associations between individual predictors and intensity-specific physical activity outcomes [13]. School-level predictors of physical activity may differ for moderate physical activity (MPA) and vigorous physical activity (VPA) [14] but there is limited evidence on factors from other domains of the socio-ecological model [12,15]. Therefore examining individual-level predictors of MPA and VPA separately could help us identify more modifiable determinants which could inform physical activity promotion. In addition, this information could be useful for health promotion programs aiming to target specific activity intensities, for example VPA which appears to decline more rapidly than other activity intensities during adolescence [13] and may be more important than lower intensity activity for weight control [16,17]. Taken together, this supports the relevance of an explorative study investigating predictors of MPA and VPA separately from overall physical activity and combined moderate and vigorous physical activity.

Differential physical activity declines have been identified for weekdays and weekend days in 10-11 year-old children [4] and there is some evidence that cross-sectional correlates of physical activity also differ for weekdays and weekend days [18]. It appears logical that influences on children’s physical activity may differ on school days compared to non-school days, however, most research investigating predictors of objectively-measured physical activity in children uses average values over all days of measurement [12]. This could limit the identification of predictors specific to weekday or weekend physical activity which could be important to successfully target physical activity promotion interventions during either of these times.

We aim to test the hypothesis that psychological, socio-cultural and environmental factors will be differentially associated with one-year change in MPA and VPA on weekends and weekdays in 9–10 year-old British children.

## Methods

### Study design and setting

The SPEEDY study is a population-based longitudinal cohort study, investigating factors associated with physical activity and dietary behaviour in 9-10 year-old (Year 5) children attending schools in the county of Norfolk, UK [19]. Ethical approval for the whole study was obtained from the University of East Anglia research ethics committee.

### Participants

Full details on participant recruitment and study procedures for the SPEEDY study baseline data collection have been described elsewhere [19]. Briefly, schools in Norfolk were purposively sampled to achieve urban and rural heterogeneity. From 227 eligible schools (those with 12 Year 5 children), 157 were approached and 92 schools were recruited. All Year 5 children (n = 3619) at the 92 schools were invited to participate. Researchers attended each school to introduce the study and distribute information packs for children and their parents. In total, 2064 children provided parental consent to participate and were measured at baseline (57% response rate).

### Data collection procedures

Baseline data collection took place during term between April and July 2007. Trained research assistants visited schools to take physical measurements, administer child questionnaires, fit accelerometers and distribute a home pack (containing an accelerometer diary, instruction sheet, questionnaire and food diary) to each child. Participants were asked to return the home packs to school one week later.

Follow-up data collection took place one year later (April and July 2008). Study information sheets and consent forms were mailed to all 2064 initial participants. Those consenting were mailed an accelerometer and a detailed instruction sheet asking participants to wear the monitor for one week and return it by mail using an addressed pre-paid envelope. Individual participants were measured at approximately the same time of year as at baseline.

### Physical activity measurement

At both baseline and follow-up, physical activity was objectively assessed using the Actigraph accelerometer (Model GT1M). The Actigraph has been shown to accurately assess energy expenditure in European children during free-living conditions [20,21]. The monitor was set to record at 5-second epochs. Children were asked to wear the monitors during waking hours for 7 days and to remove them whilst bathing, showering and swimming.

### Physical activity data processing

Accelerometry data were analyzed using a batch processing program (MAHUffe: contact corresponding author for information) to remove any data recorded after 11 pm and before 6 am. Periods of ten minutes or more that had continuous zero activity counts [22-24] and any days with less than 500 minutes of recording [23] were excluded. To maximize participant inclusion it was not necessary to have both weekend-day and weekday data at both time points. A minimum of 2 days of weekday data and 1 day of weekend data was required for inclusion in the weekday and weekend analyses respectively. The data was summarized overall and separately for weekdays and weekend days as time spent in MPA and VPA. Thresholds of 2000 cpm and 4000 cpm were used to define time in MPA and VPA, respectively [25,26]. These data were then standardized to account for potential differences in wear time between baseline and follow-up and presented as a percentage of the day, for example: change in MPA = ((follow-up MPA mins/follow-up worn time)*100) - ((baseline MPA mins/baseline worn time)*100).

### Potential predictors

All potential predictors were assessed at baseline and are described in detail in Table 1. Briefly, 38 potential predictors from the biological and socio-demographic (age, sex, BMI z-score, parent education, index of multiple deprivation), psychological (physical activity preference, self-efficacy for overcoming barriers and support seeking, personal barriers, lack of physical activity equipment), socio-cultural (peer support, family encouragement, family logistic support, physical activity and sedentary restrictions, family cohesiveness, freedom to play, games console at home, electronic equipment in the bedroom), behavioural (active travel to school) and environmental (living in a cul-de-sac, effective walkable area, woodland percentage, land use mix, perceived availability of parks and distances to green space, sports venues and school) domains were investigated. Decisions regarding recoding were made *a priori* and based on the distribution of the variables. The selection of exposure variables was informed by previous cross-sectional results from the SPEEDY study, systematic reviews of correlates of physical activity and a systematic review of predictors of change in physical activity [12,27-29]. The baseline child questionnaire was administered by researchers during school measurement sessions. Details about the questionnaire items including coding are included in Table 1; where possible the reference to the original version of the question is provided. A completed parent questionnaire was returned for 93% of participants. GIS (Geographical Information System) measurements were calculated using ArcGIS Network Analyst Version 9.2, based on the parent-reported full postal addresses of the participants. These addresses were geo-referenced for their precise location using the Ordnance Survey Address Layer 2 product. Neighbourhoods were defined as the area within a 10 minute walk (approximately 800 m) along the road network from each individual address [30]. Details about the derivation of GIS items are included in Table 1.

**Table 1:** Descriptive characteristics of potential predictors of change in physical activity

**Factor**	**Description**	**Device**	**Mean (SD) or %**
***Biological and socio-demographic***			
Age (years)	Child-reported	Child Q	9.8 (0.4)
Sex (% male)	Child-reported	Child Q	41.5%
BMI z-score	Height was measured to the nearest millimetre (Leicester height measure, Chasmors Ltd., Leicester, UK). A non-segmental bio-impedance scale was used to measure weight (to the nearest 0.1 kilogram) and impedance in light clothing (Tanita, type TBF-300A. Tokyo, Japan). Height and weight were used to calculate body mass index (BMI, kg/m^2^). British reference data was used to calculate z scores [31]	Measured	0.3 (1.1)
Parental education (% >16 years)	Parents self-reported their age at leaving full time education (reported as 3 categories and dichotomized into ≤16 years, and >16 years of age)	Parent Q	52.0%
Index of Multiple Deprivation	Computed using parent-reported home postcode. This measure combines information on deprivation from seven domains: income, employment, health, education, barriers to housing and services, living environment and crime. The derivation of this measure is described in detail elsewhere [32] and quartiles were derived for use in this analysis.	GIS	15.4 (9.5)
***Psychological factors***			
PA preference (% with preference for PA)	Sum of 4 questions asking: I would prefer to: play indoors or outdoors; walk or watch TV, run or walk; watch TV or run. Active and inactive options coded 1 and 0 respectively. Dichotomised with ≤2 as preference for inactivity; ≥3 as preference for PA recoded as 0 (reference category) and 1 respectively. [33]	Child Q	69.3%
Self efficacy in overcoming barriers (% high)	Child answered Yes (1) or No (0) to: I can do something active even: if it is hot or cold outside; if I have a lot of homework; no matter how tired I feel. Dichotomised as low self-efficacy if score ≤2, high self-efficacy if scoring 3; recoded as 0 (reference category) and 1 respectively. [34]	Child Q	42.7%
Self-efficacy in support seeking (% high)	Child answered Yes (1) or No (0) to: I can ask my parent to: sign me up for PA; my parent to do PA with me; my best friend to do something active with me. Dichotomised as low self-efficacy if score ≤2, high self-efficacy if scoring 3; recoded as 0 (reference category) and 1 respectively. [34]	Child Q	77.8%
Personal barriers (% with barriers)	Child answered Yes (1) or No (0) to: Are you ever stopped from doing PA because: there you want to watch TV; you don’t think you’re good at PA; you don’t like PA; and you might get hurt. Responses were summed and dichotomised to 0 (reference category) reporting no barriers and those reporting ≥1 barriers as having personal barriers (1). [35,36]	Child Q	52.8%
Lack of PA equipment	Child answered Yes (1) or No (0) to: Are you ever stopped from doing PA because you don’t have the equipment you need? [35,36]	Child Q	25.0%
***Socio-cultural factors***			
Peer support (% reporting peer support)	Child answered Yes (1) or No (0) to: During a typical week, do the following things happen: my friends do PA with me; I ask friends to do PA with me; My friends ask me to do PA with them. Responses were summed and then recoded as 0 no peer support and ≤1 as peer support. [35]	Child Q	69.5%
Parental encouragement	Child answered 1 (Never), 2 (once/twice/week), 3 (nearly daily), 4 (everyday) to: During a normal week, someone in my family: encourages me to do PA, tells me I am doing well at PA, tells me PA is good for my health. Responses were summed range 3–12. [37]	Child Q	8.3 (2.4)
Parental logistic support	Child answered 1 (Never), 2 (once/twice/week), 3 (nearly daily), 4 (everyday) to: During a normal week, someone in my family: does PA with me, takes me somewhere to do PA, watches me do PA. Responses were summed range 3–12. [18,37]	Child Q	6.5 (2.0)
Sedentary restrictions	Parents reported how often they restrict their child watching TV, playing computer games and using the computer with response categories N/A (0), never (0), rarely (1), sometimes (1), often (3) and very often (4). Responses were summed with range 0–12. [36]	Parent Q	6.3 (2.7)
PA restrictions	Parents reported how often they restrict their child playing outside or walking/cycling to a friends house with response categories N/A (0), never (0), rarely (1), sometimes (1), often (3) and very often (4). Responses were summed with range 0–8. [36]	Parent Q	1.7 (1.6)
Family cohesiveness (times/week)	Parents reported the number of times/week they do the following activities together as a family: eating meals, reading, sport, visiting family/friends, going to the park, swimming, cycling, watching TV, cooking with response categories as 0, 1–4 and >4 recoded as 0, 2.5 and 4.5 respectively and then summed with range 5–40.5. [36]	Parent Q	20.5 (5.1)
Freedom to play (% allowed)	Parents reported how often they allow their child to play outside anywhere within the neighbourhood with response categories never (0), rarely (1), sometimes (1), often (3) and very often (4). Dichotomised as ‘Not allowed’ (never & rarely) and ‘Allowed’ (sometimes, often and very often).	Parent Q	44.5%
Games console at home (% yes)	Children reported whether they had a games console at home with responses as Yes (1) and No (0) [35]	Child Q	88.5%
Electronic equipment in the bedroom (% yes)	Children reported whether they had a TV, PC or games console in their bedroom with responses as Yes (1) and No (0) which were summed (range 0–3) and dichotomised into (0 reference category) no media in bedroom and (1) media in bedroom. [35]	Child Q	75.0%
***Behavioural factors***			
Travel mode to school (% active)	Children reported how they usually travel to school. Car and Bus/train were recoded as 0 (passive) and walk and cycle as 1 (active). [38]	Child Q	49.8%
***Environmental factors***			
Living in a cul-de-sac (% yes)	GIS was used to determine whether the home address was a cul-de-sac, coded as 0 no (reference category) and 1 yes.	GIS	31.1%
Effective walkable area	Measure of neighbourhood connectivity. Calculated by dividing the total neighbourhood area (the area reached via the street network within 800 m from the home) by the potential walkable area (the area generated using a circular buffer with a radius of 800 m from the home). Higher values indicate higher walkability [38] (GIS-derived)	GIS	0.42 (0.16)
Woodland percentage (log%)	The percentage of participant’s neighbourhood covered by woodland or green space. Logged due to skewness.	GIS	1.26 (0.95)
Herfindahl-Hirschmann Index (HHI)	Land use mix in the participant’s neighbourhood, calculated as ∑(landusepercentage)^2^. A high HHI indicates low variation in land cover (presented higher and lower split by median) [38].	GIS	
*- Higher land use mix*			46.1%
*- Lower land use mix*			53.9%
Distance to green space (log km)	Distances (in km) to nearest green space (excluding parks), calculated as the shortest route from home address via the street network, assuming use of the nearest entrance. Logged due to skewness.	GIS	7.2 (1.0)
Distance to sport venue (log km)	Distances (in km) to nearest sport venue, calculated as the shortest route from home address via the street network, assuming use of the nearest entrance. Logged due to skewness.	GIS	6.3 (0.9)
Distance to school (log km)	Distances (in km) to school, calculated as the shortest route from home address via the street network, assuming use of the nearest entrance. Logged due to skewness.	GIS	7.2 (1.2)
Availability of parks (% yes)	Child reported perceived availability of parks. Children answered yes (1) or no (0) to: There are playgrounds, parks, or sports halls close to my home that I can use. [35]	Child Q	79.1%

### Statistical analysis

Differences in baseline characteristics by gender and between children with and without follow-up physical activity data were tested using Students t-tests or chi^2^-square tests for continuous and categorical variables, respectively. Differences in physical activity between baseline and follow-up were assessed using paired t-tests.

Simple associations between change in physical activity and potential predictors were assessed using multi-level multiple linear regression, to allow for clustering at the school level. Analyses were carried out separately for weekday and weekend days for change in time spent in MPA and change in time spent in VPA. Variables that reached p < 0.10 in the simple analyses were included in multiple multilevel linear regression models for the relevant outcome. Variables were subsequently manually removed one at a time if they did not reach p < 0.05, starting with the variable with the highest p-value. All analyses were adjusted for sex due to sex differences seen previously [4]. All models were adjusted for baseline outcome variable as the aim was to determine whether predictors could be detected independent of baseline physical activity levels [39]. Analyses were carried out using Stata 11.0 (Statacorp, College Station, TX).

## Results

Of the 2064 original SPEEDY participants invited to take part in the follow-up measurements, 1267 (61.4%) responded, of which 1019 (49.4% of original sample) consented to take part. Of these, 954 (93.6%) returned an activity monitor containing data. Valid data on change in weekday PA was obtained for 854 volunteers (41.4% of original sample) and for 718 volunteers (34.8% of original sample) for weekend analyses (1 weekend day of 500 minutes at both baseline and follow-up). Those included in this analysis were more likely to be girls (p = 0.01), to have a higher BMI z-score (p < 0.01) and to be of higher SES (all SES indicators, p < 0.01) compared to the remainder of the sample at baseline. No statistically significant differences were observed in baseline physical activity level.

Table 1 presents the baseline characteristics of the 875 participants included in one or both weekday and weekend analyses for each potential predictor. Participants were 9.8 (0.4) years-old at baseline and 41.5% male.

Table 2 shows that MPA and VPA declined significantly over one year on weekends but not on weekdays. When expressed as absolute values, MPA declined by 4.6 (21.58) minutes and VPA by 2.1 (20.1) minutes over one year.

**Table 2:** Time spent in moderate and vigorous physical activity for weekdays and weekend days

	**Baseline**	**Follow-up**	**Change**	**P value for change**
***Weekday ***(N = 854)				
MPA (minutes)	48.4 (13.5)	48.2 (14.4)	−0.2 (13.1)	P = 0.64
VPA (minutes)	24.9 (11.9)	24.5 (11.9)	−0.4 (11.2)	P = 0.25
MPA (% day)	6.6 (1.7)	6.5 (1.8)	−0.01 (1.65)	P = 0.84
VPA (% day)	3.4 (1.6)	3.3 (1.6)	−0.05 (1.47)	P = 0.29
***Weekend*** (N = 718)				
MPA (minutes)	50.1 (19.0)	45.2 (19.1)	−4.6 (21.8)	P < 0.001
VPA (minutes)	25.7 (18.5)	23.5 (17.2)	−2.1 (20.1)	P = 0.005
MPA (% day)	7.2 (2.6)	6.6 (2.6)	−0.58 (3.0)	P < 0.001
VPA (% day)	3.7 (2.7)	3.4 (2.4)	−0.27 (2.9)	P = 0.014

Table 3 shows the simple associations between potential predictors and change in physical activity. Other than for gender and baseline PA, few consistent associations were apparent across intensities and weekdays/weekends. The results of the multi-level models are shown in Table 4. Compared to boys and those with lower baseline activity, girls and those with higher baseline activity levels experienced greater decreases in MPA and VPA on both weekdays and weekends. All other predictors differed between outcome variables, for either intensity and/or day of the week. Those reporting more parental logistic support decreased their weekend MPA and VPA less than those reporting less parental logistic support. However, participants reporting higher peer support were less likely to experience declines in weekday MPA and VPA.

**Table 3:** Simple associations between predictors and change in moderate and vigorous physical activity for weekdays and weekends

	***Weekday *(N = 854)**		***Weekend day *(N = 718)**	
	**% change MPA**	**% change VPA**	**% change MPA**	**% change VPA**
	**β (CI 95%)**	**β (CI 95%)**	**β (CI 95%)**	**β (CI 95%)**
**Biological and socio-demographic factors**				
Age (y)	0.22 (−0.01, 0.45)^#^	0.02 (−0.22, 0.26)	0.20 (−0.21, 0.62)	−0.32 (−0.80, 0.16)
Sex (ref: boys)	−0.53 (−0.79, -0.27)^***^	−0.03 (−0.04, -0.02)^***^	−0.67 (−1.10, -0.24)^**^	−0.64 (−1.06, -0.22)^**^
BMI z-score	−0.03 (−0.14, 0.09)	0.02 (−0.06, 0.09)	−0.04 (−0.20, 0.13)	−0.27 (−0.44, -0.10)^**^
Parent education (high vs. low)	−0.22 (−0.46, 0.01)^#^	−0.23 (−0.47, 0.02)^#^	−0.22 (−0.61, 0.17)	−0.01 (−0.32, 0.29)
IMD	−0.01 (−0.02, 0.01)	−0.01 (−0.01, 0.01)	0.016 (−0.01, 0.04)	0.17 (−0.01, 0.04)
**Psychological factors**				
PA preference (high vs. low)	0.06 (−0.16, 0.28)	−0.04 (−0.23, 0.15)	0.56 (0.18, 0.93)^**^	0.48 (0.15, 0.81)^**^
SE for overcoming barriers (high vs. low)	0.09 (−0.11, 0.30)	−0.05 (−0.25, 0.15)	0.38 (0.008, 0.75)^*^	0.11 (−0.23, 0.44)
SE for support seeking (high vs. low)	−0.03 (−0.27, 0.22)	0.02 (−0.21, 0.26)	−0.05 (−0.47, 0.37)	0.14 (−0.25, 0.54)
Personal barriers (high vs. low)	−0.05 (−0.26, 0.16)	0.10 (−0.08, 0.28)	−0.11 (−0.50, 0.27)	−0.23 (−0.57, 0.11)
Home PA equipment (high vs. low)	0.04 (−0.20, 0.28)	0.31 (0.08, 0.54)^**^	−0.63 (−1.10, -0.17)^**^	−0.28 (−0.66, 0.09)
**Socio-cultural factors**				
Peer support (high vs. low)	0.17 (0.01, 0.33)^*^	0.22 (0.05, 0.38)^**^	0.35 (−0.013, 0.72)^#^	0.25 (−0.12, 0.62)
Family encouragement	0.01 (−0.03, 0.06)	0.01 (−0.04, 0.05)	0.02 (−0.06, 0.10)	0.07 (−0.10, 0.14)^#^
Family logistic support	0.03 (−0.02, 0.08)	0.01 (−0.06, 0.06)	0.18 (0.09, 0.27)^***^	0.18 (0.08, 0.29)^***^
Sedentary restrictions	0.01 (−0.04, 0.05)	0.03 (−0.01, 0.06)	0.02 (−0.05, 0.08)	−0.03 (−0.10, 0.03)
PA restrictions	0.01 (−0.06, 0.08)	0.01 (−0.05, 0.07)	−0.03 (−0.016, 0.10)	−0.05 (−0.17, 0.07)
Family cohesiveness	−0.01 (−0.02, 0.02)	−0.01 (−0.03, 0.01)	0.015 (−0.03, 0.05)	0.04 (−0.01, 0.07)^#^
Freedom to play (yes vs. no)	−0.01 (−0.22, 0.20)	0.08 (−0.14, 0.30)	0.01 (−0.33, 0.35)	−0.27 (−0.57, 0.04)^#^
Games console at home (yes vs. no)	0.32 (−0.01, 0.63)^#^	0.06 (−0.04, -0.02)^***^	−0.14 (−0.78, 0.50)	−0.02 (−0.61, 0.57)
Electronic equipment in bedroom (yes vs. no)	0.27 (0.02, 0.52)^**^	0.15 (−0.04, 0.34)	0.13 (−0.29, 0.55)	0.12 (−0.32, 0.57)
**Behavioural factor**				
Active Travel to school (active vs. passive)	0.10 (−0.13, 0.33)	0.06 (−0.11, 0.34)	0.06 (−0.31, 0.43)	−0.17 (−0.48, 0.14)
**Environmental factors**				
Living in a cul de sac (yes vs. no)	−0.22 (−0.47, 0.02)^#^	0.08 (−0.15, 0.30)	−0.17 (−0.55, 0.20)	0.06 (−0.27, 0.39)
Effective walkable area	−0.57 (−0.37, 0.23)	−0.49 (−1.09, 0.12)	−0.27 (−1.5, 0.97)	0.13 (−1.05, 1.30)
Woodland percentage (log%)	−0.05 (−0.17, 0.07)	0.04 (−0.06, 0.14)	0.04 (−0.14, 0.23)	0.15 (−0.02, 0.32)^#^
Land use mix (Lower v. higher)	−0.28 (−0.51, -0.05)^**^	−0.16 (−0.36, 0.04)	−0.31 (−0.68, 0.05)^#^	0.11 (−0.26, 0.48)
Distance to green space (log km)	−0.01 (−0.14, 0.11)	−0.01 (−0.12, 0.10)	−0.08 (−0.26, 0.10)	−0.04 (−0.19, 0.12)
Distance to sport venue (log km)	0.05 (−0.06, 0.17)	0.04 (−0.05, 0.14)	0.14 (−0.09, 0.36)	0.07 (−0.12, 0.27)
Distance to school (log km)	−0.09 (−0.18, -0.01)^*^	−0.10 (−0.18, -0.02)^**^	0.06 (−0.09, 0.22)	0.03 (−0.16, 0.21)
Availability of parks (yes vs. no)	0.18 (−0.06, 0.43)	0.08 (−0.20, 0.36)	0.10 (−0.39, 0.59)	−0.05 (−0.57, 0.47)

Abbreviation: *PA*, Physical activity; *SE*, self-efficacy; *MPA*, Moderate physical activity; *VPA*, Vigorous physical activity.

Results from simple multivariable mixed effects multilevel regression models adjusted for sex, baseline physical activity and school clustering.

P values: ^***^p < 0.001; ^**^p < 0.02 to p = 0.001; ^*^p = 0.02 to p < 0.05; ^#^p = 0.05 to p = 0.10.

**Table 4:** Final adjusted associations between predictors and change in moderate and vigorous physical activity for weekdays and weekends

		**Β**	**CI 95%**	**p value**
***Weekday *(N = 854)**				
**MPA**				
Baseline MPA		−0.05	−0.06, -0.05	<0.001
Sex	Boys			
	Girls	−0.53	−0.78, 0.28	<0.001
HHI (Land use mix)	Lower			
	Higher	0.29	−0.51, -0.06	0.014
Peer support	Low			
	High	0.18	0.02, 0.34	0.028
**VPA**				
Baseline VPA		−0.03	−0.04, -0.02	<0.001
Sex	Boys			
	Girls	−0.45	−0.66, -0.23	<0.001
Distance to school (log)		−0.10	−0.18, -0.01	0.021
PA equipment	Low			
	High	0.33	0.09, 0.57	0.007
Peer support	Low support			
	High support	0.22	0.06, 0.38	0.007
***Weekend day *(N = 718)**				
**MPA**				
Baseline MPA		−0.09	−1.0, -0.08	<0.001
Sex	Boys			
	Girls	−0.63	−1.02, -0.02	0.001
PA preference	No preference			
	Having preference	0.46	0.06, 0.86	0.02
Family logistic support		0.15	0.05, 0.25	0.003
**VPA**				
Baseline VPA		−0.10	−0.12, 0.09	<0.001
Sex	Boys			
	Girls	−0.61	−1.04, -0.19	0.004
BMI z-score		−0.28	−0.45, -0.11	0.001
Family logistic support		0.19	0.09, 0.29	<0.001

Abbreviation: *PA,* Physical activity; *MPA*, Moderate physical activity; *VPA*, Vigorous physical activity.

Results from final multivariable mixed effects multilevel regression models adjusted for sex, baseline physical activity and for school clustering.

## Discussion

Objectively-measured MPA and VPA declined on weekends but not weekdays over one year in British children. Higher baseline physical activity and being a girl were consistent predictors for greater declines in MPA and VPA on both weekdays and weekends. Other predictors differed across activity intensity and/or day of the week and included those from multiple domains of the socio-ecological model. Participants reporting higher parental logistic support experienced less of a weekend decline in MPA and VPA and those reporting peer support for activity had less of a weekday decline in MPA and VPA.

There is little data available with which to compare our results as few studies have examined predictors of change in physical activity using objective data and fewer have stratified analyses for MPA and VPA on weekdays and weekends [11,12]. A review of predictors of change in physical activity only identified previous physical activity, self-efficacy, perceived behavioural control and intention as consistent predictors of change in overall physical activity among 10-13 year-old children [12]. Current results showed previous physical activity level to be associated with all four outcome variables used here. An association with self-efficacy for overcoming barriers was only found in the simple model for weekend MPA [12]. Neither perceived behavioural control nor intention to become physically active was measured here. Nevertheless, our findings of largely different predictors for MPA and VPA on weekdays and weekends support the relevance of investigating predictors of change in more specific outcome variables than overall physical activity or combined moderate and vigorous physical activity.

Aside from supporting the investigation of predictors of physical activity separately by activity intensity and weekends and weekdays, this study has provided some potential intervention targets. Participants reporting greater peer and parent support for physical activity experienced less of a decline in weekday and weekend physical activity respectively. The association with family support contrasts the findings of no association in longitudinal research [40-42] but supports cross-sectional evidence [28], although this is the first study differentiating the associations for different times of the week. Perhaps the lack of consensus in the literature may be partly due to the relevance of different support roles at specific times of the week. Our results appear logical in that parent support may be more relevant at weekends when children are more likely to be with their family, whereas peer support is more relevant on weekdays.

Participants reporting the highest tertile of parent support experienced a weekend VPA decline of approximately 1.9 minutes less than participants in the lowest group of parent support. This is equivalent to 7% of total baseline VPA and it is therefore likely that increasing parent or peer support for physical activity may be appropriate only as one component of a more complex physical activity promotion intervention. Although our results indicate that both parent and peer support may be important for preventing physical activity declines during late childhood it is currently unknown what is the best way to engage parents in youth physical activity promotion [43]. In addition, these children are about to enter adolescence and it is also uncertain to what extent the importance of peer support surpasses family support for physical activity as children age [44]. Nevertheless, our results suggest that physical activity interventions in late childhood may benefit from targeting weekend physical activity by increasing parental involvement, specifically logistic support, which includes being active with the child, providing transport and watching them do activity.

Previous data from this cohort indicated that participants with a higher body fat percentage had a greater decline in MVPA [4]. In the current analyses BMI z-score only remained in the final model for weekend VPA. Other evidence regarding the association between anthropometry and physical activity declines among children is inconclusive [45-50]. This lack of consensus and our varying results could be due to differential associations between different anthropometry indicators and intensity-specific physical activity outcomes. As a higher baseline BMI z-score appears to be associated with a greater decline in weekend VPA, this perhaps partly supports recent suggestions that VPA may be more important than lower intensity activity for weight control [16,17], and that body composition predicts changes in activity more strongly than the reverse [2,51]. An examination of both physical activity and anthropometric measures at baseline and follow-up would be necessary to further examine these associations. This may suggest that investigating predictors of VPA separately from combined moderate and vigorous physical activity may be particularly relevant for physical activity promotion with the aim of obesity prevention and control.

### Strengths and limitations

This study builds on the relatively small but expanding body of longitudinal high quality research on predictors of physical activity change. Further, these results indicate that predictors of change in physical activity appear to differ for MPA and VPA and over weekdays and weekends. This well-characterized large cohort allows for investigation of a wide selection of predictors of change with accurately measured exposures and stratified outcome variables. Accelerometry data should more accurately represent physical activity than a questionnaire but is not able to accurately assess some activities such as swimming and cycling [52]. The majority of potential determinants investigated have been used previously; however, some of these were developed for this study. Although all questions were pilot tested in the appropriate age group prior to use, we must acknowledge that while it is unlikely, it is possible that respondents could attribute a different meaning to some of the items than we do. Only 49.4% of the baseline population consented to participate with boys and children of lower SES more likely to be excluded from analyses. Although no differences were observed for baseline physical activity levels, the differential drop-out limits the generalisability of our observations. The weekday data used here includes both school time and out of school time and we are not able to disentangle how much of the decline occurs at school and how much is outside school. Previous examination of hourly physical activity change in this cohort indicated greater declines during free time [4], therefore it is likely that the majority of the decline occurs out of school. There is controversy as to the best way to address the analysis of change, however, adjusting for baseline levels allowed us to examine potential predictors while assuming the same initial physical activity level and was considered appropriate to address regression to the mean [39]. We acknowledge the large number of statistical tests in this exploratory study and individual results should therefore be interpreted with caution.

### Implications

Longitudinal studies examining the relative contributions of MPA and VPA to the physical decline during adolescence, and how change differs over weekdays and weekends, could be valuable to better target physical activity promotion. Further, the investigation of predictors of change in physical activity specifically for MPA/VPA and weekdays/weekends could aid the design of physical activity promotion interventions. Physical activity declined more during weekends compared to weekdays so it is possible that schools may already be protective of declines which could otherwise be of the magnitude observed at weekends. Therefore our results suggest that preventing declines at weekends may have the biggest impact on physical activity maintenance and therefore investigation of predictors of change specific to weekends appears relevant. In order to target weekend physical activity, further emphasis on developing family and community based interventions and improving recruitment and retention in these challenging research domains may be especially important. These results also reinforce the importance of support from others regarding changes in children’s physical activity as parental logistic support appeared protective of a weekend decline in MPA and VPA and peer support protective against a weekday decline in MPA and VPA.

## Conclusions

These results highlight the importance of investigating time- and intensity-specific predictors of change in physical activity and provide some suggestions for targeting physical activity promotion interventions accordingly. In addition to continued focus on school PA promotion, more effort to target interventions during weekends, such as in the family and community appears important. Focusing on peer support to encourage weekday physical activity and parent support during weekends could be important targets as part of more complex physical activity promotion programs.

## Abbreviations

Cpm: Counts per minute; GIS: Geographical information systems; Mins: Minutes; MPA: Moderate physical activity; SPEEDY: Sport, Physical activity and Eating behaviour: Environmental Determinants in Young people; PA: Physical activity; VPA: Vigorous physical activity.

## Competing interests

The authors declare that they have no competing interests.

## Authors’ contributions

KC supervised and coordinated follow-up data collection, carried out the analyses, drafted the manuscript, critically reviewed the manuscript, and approved the final manuscript as submitted. CC contributed to initial analyses and drafting of the initial manuscript and approved the final manuscript as submitted. AJ, SJ and UE were involved with the conceptualization and design of the initial study, reviewed and revised the manuscript, and approved the final manuscript as submitted. EvS conceptualized and designed the study, designed the data collection instruments, and coordinated baseline data collection, critically reviewed the manuscript, and approved the final manuscript as submitted.

## Authors’ information

Kirsten Corder are Christopher Craggs are joint first author.

## References

[B1] Janssen I Leblanc AG Systematic review of the health benefits of physical activity and fitness in school-aged children and youth Int J Behav Nutr Phys Act 2010 7 40 10.1186/1479-5868-7-40 20459784 PMC2885312

[B2] Ekelund U Luan J Sherar LB Esliger DW Griew P Cooper A Moderate to vigorous physical activity and sedentary time and cardiometabolic risk factors in children and adolescents JAMA 2012 307 704 712 10.1001/jama.2012.156 22337681 PMC3793121

[B3] Smith A Biddle S Youth physical activity and sedentary behavior 2008 Champaign, IL: Human Kinetics

[B4] Corder K van Sluijs EM Ekelund U Jones AP Griffin SJ Changes in children’s physical activity over 12 months: longitudinal results from the SPEEDY study Pediatrics 2010 126 e926 e935 10.1542/peds.2010-0048 20837590

[B5] Dumith SC Gigante DP Domingues MR Kohl HW 3rd Physical activity change during adolescence: a systematic review and a pooled analysis Int J Epid 2011 40 685 698 10.1093/ije/dyq272 21245072

[B6] Telema R Yang X Viikari J Valimaki I Wanne O Raitakari O Physical activity from childhood to adulthood a 21-year tracking study Am J Prev Med 2005 28 267 273 10.1016/j.amepre.2004.12.003 15766614

[B7] Khaw K-T Wareham N Bingham S Welch A Luben R Day N Combined impact of health behaviours and mortality in Men and women: the EPIC-norfolk prospective population study PLoS Med 2008 5 e12 10.1371/journal.pmed.0050012 18184033 PMC2174962

[B8] Kriemler S Meyer U Martin E van Sluijs EM Andersen LB Martin BW Effect of school-based interventions on physical activity and fitness in children and adolescents: a review of reviews and systematic update Brit J Sports Med 2011 45 923 930 10.1136/bjsports-2011-090186 21836176 PMC3841814

[B9] van Sluijs E McMinn A Griffin S Effectiveness of interventions to promote physical activity in children and adolescents: systematic review of controlled trials Brit Med J 2007 335 7622 703 10.1136/bmj.39320.843947.BE 17884863 PMC2001088

[B10] van Sluijs EM Kriemler S McMinn AM The effect of community and family interventions on young people’s physical activity levels: a review of reviews and updated systematic review Brit J Sports Med 2011 45 914 922 10.1136/bjsports-2011-090187 21836175 PMC3736309

[B11] Corder K Ogilvie D van Sluijs EM Invited commentary: physical activity over the life course–whose behavior changes, when, and why? Am J Epid 2009 170 1078 1081 discussion 1082–1073 10.1093/aje/kwp273 PMC3672467 19767350

[B12] Craggs C Corder K van Sluijs EM Griffin SJ Determinants of change in physical activity in children and adolescents: a systematic review Am J Prev Med 2011 40 645 658 10.1016/j.amepre.2011.02.025 21565658 PMC3100507

[B13] Corder K Sharp S Atkin A Griffin S Jones A Ekelund U van Sluijs E Change in objectively measured physical activity during the transition to adolescence: targets for intervention Under review 10.1136/bjsports-2013-093190 PMC4453714 24273308

[B14] Mantjes JA Jones AP Corder K Jones NR Harrison F Griffin SJ van Sluijs EM School related factors and 1yr change in physical activity amongst 9–11 year old English schoolchildren Int J Behav Nutr Phys Act 2012 9 153 10.1186/1479-5868-9-153 23276280 PMC3544638

[B15] Sallis J Owen N Fisher E Glanz K, Rimer B, Viswanath K Ecological models of health behaviour Health behavior and health education: theory, research and practice 2008 4 San Francisco: Jossey-Bass 465 485

[B16] Steele RM van Sluijs EM Cassidy A Griffin SJ Ekelund U Targeting sedentary time or moderate- and vigorous-intensity activity: independent relations with adiposity in a population-based sample of 10-y-old British children Am J Clin Nutr 2009 90 1185 1192 10.3945/ajcn.2009.28153 19776141

[B17] Janssen I Ross R Vigorous intensity physical activity is related to the metabolic syndrome independent of the physical activity dose Int J Epid 2012 41 1132 40 10.1093/ije/dys038 PMC3429871 22447838

[B18] McMinn AM Griffin SJ Jones AP van Sluijs EM Family and home influences on children’s after-school and weekend physical activity Eur J Public Health 2012 Apr 29. [Epub ahead of print] 10.1093/eurpub/cks160 PMC3784797 23172732

[B19] Van Sluijs E Skidmore P Mwanza K Jones A Callaghan A Ekelund U Harrison F Harvey I Panter J Wareham N Physical activity and dietary behaviour in a population-based sample of British 10-year old children: the SPEEDY study Sport, Physical activity and Eating behaviour: Environmental Determinants in Young people BMC Publ Health 2008 8 388 10.1186/1471-2458-8-388 PMC2605463 19014571

[B20] Ekelund U Åman J Westerterp K Is the ArteACC index a valid indicator of free-living physical activity in adolescents? Obes Res 2003 11 793 801 10.1038/oby.2003.110 12805401

[B21] Ekelund U Sjöström M Yngve A Poortvliet E Nilsson A Froberg K Wedderkopp N Westerterp K Physical activity assessed by activity monitor and doubly labelled water in children Med Sci Sports Exerc 2001 33 275 281 11224818 10.1097/00005768-200102000-00017

[B22] Eiberg H Hasselstrom H Gronfeldt V Froberg K Svensson J Andersen LB Maximum oxygen uptake and objectively measured physical activity in Danish children 6–7 years of age: the Copenhagen school child intervention study Brit J Sports Med 2005 39 725 730 10.1136/bjsm.2004.015230 16183768 PMC1725036

[B23] Mattocks C Ness A Leary S Tilling K Blair S Shield J Deere K Saunders J Kirkby J Davey Smith G Use of accelerometers in a large field-based study of childen: protocols, design issues, and effects on precision J Phys Act Health 2008 5 S98 S111 18364528 10.1123/jpah.5.s1.s98

[B24] Riddoch CJ Bo Andersen L Bo Andersen L Wedderkopp N Harro M Klasson-Heggebo L Sardinha LB Cooper AR Ekelund U Physical activity levels and patterns of 9- and 15-yr-old European children Med Sci Sports Exerc 2004 36 86 92 10.1249/01.MSS.0000106174.43932.92 14707773

[B25] Brage S Brage N Wedderkopp N Froberg K Reliability and validity of the computer science and applications accelerometer in a mechanical setting Meas Phys Educ Exerc Sci 2003 7 101 119 10.1207/S15327841MPEE0702_4

[B26] Trost S Ward D Moorehead S Watson P Riner W Burke J Validity of the computer and science and applications (CSA) activity monitor in children Med Sci Sports Exerc 1998 30 629 633 10.1097/00005768-199804000-00023 9565947

[B27] Sallis JF Prochaska JJ Taylor WC A review of correlates of physical activity of children and adolescents Med Sci Sports Exerc 2000 32 963 975 10795788 10.1097/00005768-200005000-00014

[B28] van der Horst K Chin A Paw M Twisk J van Mechelen W A brief review on correlates of physical activity and sedentariness in youth Med Sci Sports Exerc 2007 39 1241 1250 10.1249/mss.0b013e318059bf35 17762356

[B29] Ferreira I van der Horst K Wendel-Vos W Kremers S van Lenthe F Brug J Environmental correlates of physical activity in youth - a review and update Obes Rev 2006 8 129 154 17300279 10.1111/j.1467-789X.2006.00264.x

[B30] Panter JR Jones AP van Sluijs EM Griffin SJ Attitudes, social support and environmental perceptions as predictors of active commuting behaviour in school children J Epid Comm Health 2010 64 41 48 10.1136/jech.2009.086918 PMC3703574 19465403

[B31] 2009 World population data sheet 2009 http://www.prb.org

[B32] Noble M McLennan D Wilkinson K Whitworth A Barnes H The english indices of deprivation 2007 http://webarchive.nationalarchives.gov.uk/20120919132719/http://www.communities.gov.uk/documents/communities/pdf/733520.pdf

[B33] Sallis JF McKenzie TL Alcaraz JE Kolody B Hovell MF Nader PR Project SPARK. Effects of physical education on adiposity in children Ann N Y Acad Sci 1993 699 127 136 10.1111/j.1749-6632.1993.tb18844.x 8267303

[B34] Saunders R Pate R Felton G Dowda M Weinrich M Ward D Parsons M Baranowski T Development of questionnaires to measure psychosocial influences on children’s physical activity Prev Med 1997 26 241 247 10.1006/pmed.1996.0134 9085394

[B35] Ommundsen Y Page A Po-Wen K Cooper AR Cross-cultural, age and gender validation of a computerised questionnaire measuring personal, social and environmental associations with children’s physical activity: The European Youth Heart Study Int J Behav Nutr Phys Act 2008 5 29 10.1186/1479-5868-5-29 18489736 PMC2430583

[B36] Salmon J Timperio A Telford A Carver A Crawford D Association of family environment with Children’s television viewing and with Low level of physical activity Obes Res 2005 13 1939 1951 10.1038/oby.2005.239 16339126

[B37] Prochaska JJ Rodgers MW Sallis JF Association of parent and peer support with adolescent physical activity Res Q Exerc Sport 2002 73 206 210 10.1080/02701367.2002.10609010 12092896

[B38] Panter JR Jones AP Van Sluijs EM Griffin SJ Neighborhood, route, and school environments and children’s active commuting Am J Prev Med 2010 38 268 278 10.1016/j.amepre.2009.10.040 20171528 PMC3819023

[B39] Fitzmaurice G A conundrum in the analysis of change Nutr 2001 17 360 361 10.1016/S0899-9007(00)00593-1 11369183

[B40] Crawford D Cleland V Timperio A Salmon J Andrianopoulos N Roberts R Giles-Corti B Baur L Ball K The longitudinal influence of home and neighbourhood environments on children’s body mass index and physical activity over 5 years: the CLAN study Int J Obes 2010 34 1177 1187 10.1038/ijo.2010.57 20351728

[B41] DiLorenzo TM Stucky-Ropp RC Vander Wal JS Gotham HJ JS Determinants of exercise among children. II. A longitudinal analysis Prev Med 1998 27 470 477 10.1006/pmed.1998.0307 9612838

[B42] Duncan S Duncan T Stryker L Chaumeton N A cohort-sequential latent growth model of physical activity from ages 12 to 17 years Ann Behav Med 2007 33 80 89 10.1207/s15324796abm3301_9 17291173 PMC2729662

[B43] O’Connor TM Jago R Baranowski T Engaging parents to increase youth physical activity a systematic review Am J Prev Med 2009 37 141 149 10.1016/j.amepre.2009.04.020 19589450

[B44] Jackson C Henderson M Frank J Haw S An overview of prevention of multiple risk behaviour in adolescence and young adulthood J Publ Health 2012 34 31 40 10.1093/pubmed/fdr113 22363029

[B45] Barnett TA O’Loughlin J Paradis G One- and two-year predictors of decline in physical activity among inner-city schoolchildren Am J Prev Med 2002 23 121 128 10.1016/S0749-3797(02)00464-6 12121800

[B46] Hampson SE Andrews JA Peterson M Duncan SC A cognitive-behavioral mechanism leading to adolescent obesity: children’s social images and physical activity Ann Behav Med 2007 34 287 294 10.1007/BF02874553 18020938 PMC2424024

[B47] Kahn JA Huang B Gillman MW Field AE Austin SB Colditz GA Frazier AL Patterns and determinants of physical activity in U.S. adolescents J Adolesc Health 2008 42 369 377 10.1016/j.jadohealth.2007.11.143 18346662

[B48] Goran MI Gower BA Nagy TR Johnson RK Developmental changes in energy expenditure and physical activity in children: evidence for a decline in physical activity in girls before puberty Pediatrics 1998 101 887 891 10.1542/peds.101.5.887 9565420

[B49] Neumark-Sztainer D Story M Hannan PJ Tharp T Rex J Factors associated with changes in physical activity: a cohort study of inactive adolescent girls Arch Pediatr Adolesc Med 2003 157 803 810 10.1001/archpedi.157.8.803 12912787

[B50] Nader PR Bradley RH Houts RM McRitchie SL O’Brien M Moderate-to-vigorous physical activity from ages 9 to 15 years JAMA 2008 300 295 305 10.1001/jama.300.3.295 18632544

[B51] Kwon S Janz KF Burns TL Levy SM Effects of adiposity on physical activity in childhood: Iowa Bone Development Study Med Sci Sports Exerc 2011 43 443 448 20631643 10.1249/MSS.0b013e3181ef3b0a PMC3130563

[B52] Corder K Ekelund U Steele RM Wareham NJ Brage S Assessment of physical activity in youth J Appl Physiol 2008 105 977 987 10.1152/japplphysiol.00094.2008 18635884

